# The tumor microenvironment of cutaneous squamous cell carcinoma in high-risk patient groups: A scoping review

**DOI:** 10.1016/j.xjidi.2026.100455

**Published:** 2026-02-02

**Authors:** Wandong Wang, Clara Harrs, Maria C. Bolling, Barbara Horváth, Gilles F.H. Diercks, Emőke Rácz

**Affiliations:** 1 Department of Dermatology, University Medical Center Groningen, University of Groningen, Groningen, The Netherlands; 2 Center for Blistering Diseases, Department of Pathology, University Medical Center Groningen, University of Groningen, Groningen, The Netherlands

**Keywords:** Cutaneous squamous cell carcinoma, Epidermolysis bullosa, Organ transplant recipients, Tumor microenvironment

## Abstract

Cutaneous squamous cell carcinoma (cSCC) is a prevalent skin cancer in the general population that poses so far unresolved challenges in high-risk groups such as organ transplant recipients and individuals with recessive dystrophic epidermolysis bullosa. Although most cases of cSCC respond well to standard treatments, these 2 groups often face more aggressive disease, characterized by higher rates of metastasis and cancer-specific mortality. The tumor microenvironment plays a pivotal role in cSCC progression, influencing tumor growth, immune evasion, and therapy response. Therefore, this scoping review aims to systematically investigate how the tumor microenvironment in these high-risk cSCC differs from that of sporadic cSCC, highlight shared tumorigenic mechanisms, and identify knowledge gaps for future research. Specifically, we review immune cell infiltration, epithelial–mesenchymal transition, extracellular matrix remodeling, and related biomarkers, while also exploring potential therapeutic targets. It is surmised that both organ transplant recipients cSCC and recessive dystrophic epidermolysis bullosa cSCC may exhibit a permissive tumor microenvironment, potentially characterized by immune dysfunction and enhanced TGFβ signaling, contributing to tumor aggressiveness. Notably, organ transplant recipients cSCC primarily demonstrates immune exhaustion, whereas recessive dystrophic epidermolysis bullosa cSCC is driven by chronic tissue damage with concomitant extracellular matrix remodeling. A better understanding of tumor microenvironment features in these high-risk cSCC may help develop novel targeted therapies to improve patient outcomes.

## Graphical abstract

**Figure:**
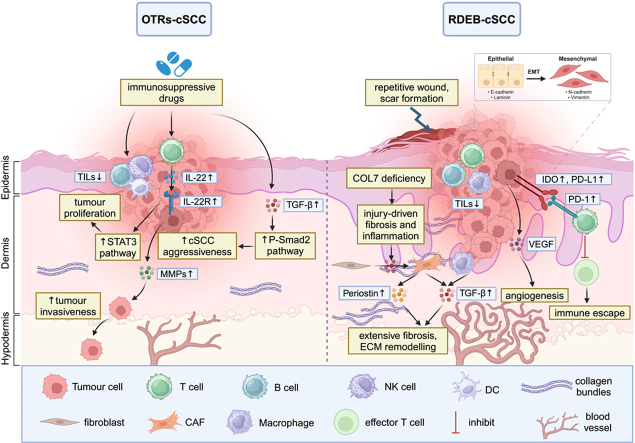

## Introduction

Cutaneous squamous cell carcinoma (cSCC) is the second most common skin malignancy in the world after basal cell carcinoma (Beebe et al, 2022). cSCC predominantly affects middle-aged and elderly people and is usually found in sun-exposed areas. Risk factors for cSCC include prolonged UV exposure, lighter skin, compromised immune system, precancerous lesions (eg, actinic keratosis [AK]), certain hereditary disorders, beta-human papillomavirus infection, and environmental carcinogens (Thai et al, 2021; Winge et al, 2023). Although most patients have favorable outcomes after surgery and/or radiotherapy, a minority (approximately 2% in immunocompetent patients and up to 6% or higher in immunosuppressed or high-risk groups) develop metastases, and the disease-specific mortality rates are roughly 1% and 3–4%, respectively (Corchado-Cobos et al, 2023; de Jong et al, 2024; Gjersvik et al, 2023; Thai et al, 2021).

cSCC is the most common type of cancer in organ transplant recipients (OTRs). The incidence of cSCC is 65–200 times higher in OTRs than in the general population (Bottomley et al, 2019; Massey et al, 2021), primarily owing to the long-term use of immunosuppressive drugs to prevent organ rejection, which simultaneously impairs the immune system’s ability to monitor and clear cancer cells (Corchado-Cobos et al, 2023). Several studies have reported that approximately 10% of OTRs develop cSCC within 5 years of transplantation and 40–60% at ≥10 years after transplantation (Bottomley et al, 2016; Zilberg et al, 2023).

Epidermolysis bullosa (EB), a group of hereditary skin fragility disorders characterized by the formation of blisters and wounds, is another disease susceptible to the development of high-risk cSCC (Fine et al, 2009). Depending on the level of blister formation within the skin, EB is classified in 4 main types (EB simplex, junctional EB, dystrophic EB, and Kindler syndrome) and 34 subtypes on the basis of inheritance, molecular features, and clinical characteristics. Dystrophic EB can be further subdivided into dominant dystrophic EB and recessive dystrophic EB (RDEB) (Has et al, 2020). Owing to defective type VII collagen (COL7) resulting from sequence variants in the *COL7A1* gene, RDEB is characterized by more severe clinical manifestations. A serious complication in RDEB is the formation of invasive cSCC, which is the leading cause of morbidity and death in patients with RDEB (Castelo et al, 2019; Kim et al, 2018; Robertson et al, 2021).

Compared with sporadic cSCC, cSCC in OTRs (OTRs-cSCC) and RDEB-associated cSCC (RDEB-cSCC) demonstrate increased aggressiveness with a high tendency for metastasis. However, the pathogenesis underlying this aggressive behavior remains poorly understood in both patient groups, and treatment options are limited. Although immune checkpoint inhibitors (ICIs) offer a promising approach for treating metastatic or advanced cSCC, their effectiveness in these 2 high-risk populations is significantly reduced (Koch Hein et al, 2023; Stevenson et al, 2017).

**Figure 1:**
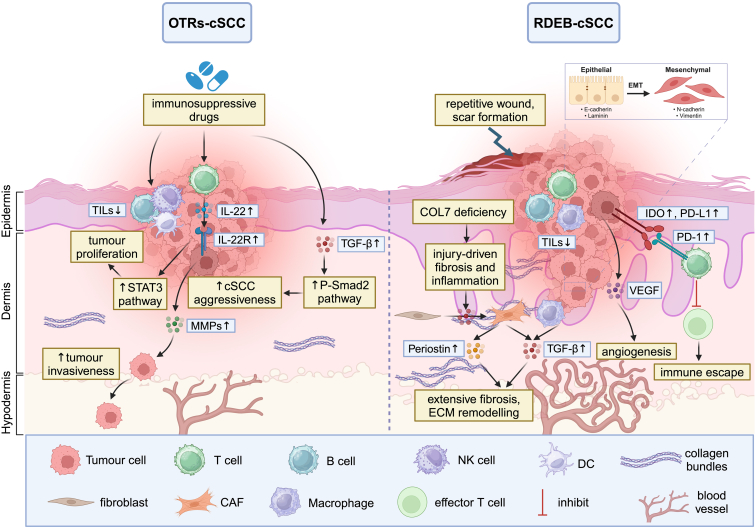
**Schematic illustration of key pathogenic pathways involved in cSCC development in OTRs and patients with RDEB.** OTRs-cSCC (left) is driven by long-term use of immunosuppressive drugs, leading to reduced TILs, increased IL-22/IL-22R signaling, STAT3 pathway activation, and enhanced tumor invasiveness through MMPs upregulation. Increased TGFβ signaling in the TME of OTRs-cSCC contributes to stromal modifications and cancer progression through P-Smad2 activation. In RDEB-cSCC (right), chronic wound healing due to COL7 deficiency leads to fibrosis, ECM remodeling, and a permissive TME. Elevated TGFβ, periostin, VEGF, and immune checkpoint markers (IDO, PD-L1, PD-1) contribute to tumor progression, angiogenesis, and immune escape. Both high-risk cSCC types share features of immune dysregulation and TGFβ-driven stromal remodeling, offering potential therapeutic targets. COL7, type VII collagen; cSCC, cutaneous squamous cell carcinoma; ECM, extracellular matrix; IDO, indoleamine 2,3-dioxygenase; MMP, matrix metalloproteinase; OTR, organ transplant recipient; OTRs-cSCC, cutaneous squamous cell carcinoma in organ transplant recipient; P-Smad2, phosphorylated Smad2; RDEB, recessive dystrophic epidermolysis bullosa; RDEB-cSCC, recessive dystrophic epidermolysis bullosa–cutaneous squamous cell carcinoma; STAT3, signal transducer and activator of transcription 3; TIL, tumor-infiltrating lymphocyte; TME, tumor microenvironment.

There is increasing knowledge that the composition of the tumor microenvironment (TME) can have a crucial impact on tumor growth and the development of metastasis and therapy resistance in sporadic cSCC (Amôr et al, 2021; Saeidi et al, 2023). The TME is a complex ecosystem surrounding cancer cells, comprising various noncancerous cells embedded within an altered extracellular matrix (ECM). These components include diverse immune cells, cancer-associated fibroblasts (CAFs), endothelial cells, other tissue-resident cell types and some noncellular components (eg, cytokines or chemokines) (de Visser and Joyce, 2023). In this context, it is hypothesized that a permissive TME may also play a critical role in driving the development of aggressive OTRs-cSCC and RDEB-cSCC, specifically through immune dysregulation and ECM remodeling (Küttner et al, 2013; Zilberg et al, 2023) (Figure 1). We surmise that the TME of these 2 clinically aggressive cSCC types have a similar composition facilitating cancer progression and metastasis, despite diverging etiologies. There is currently no existing literature review comparing findings about the TME of OTRs-cSCC and RDEB-cSCC, but more knowledge about the TME of both high-risk cSCC groups could help to detect important protumorigenic signaling pathways and potential biomarkers for novel therapeutic approaches, such as immune checkpoint molecules.

Therefore, the aim of this scoping review was to systematically investigate in which aspects the TME of these 2 high-risk cSCC differ compared with that of sporadic cSCC. Moreover, we aimed to highlight potential shared tumorigenic mechanisms in OTRs-cSCC and RDEB-cSCC, emphasize knowledge gaps for further research, and provide insights into promising therapeutic targets for both high-risk groups in the future.

## Results

**Figure 2:**
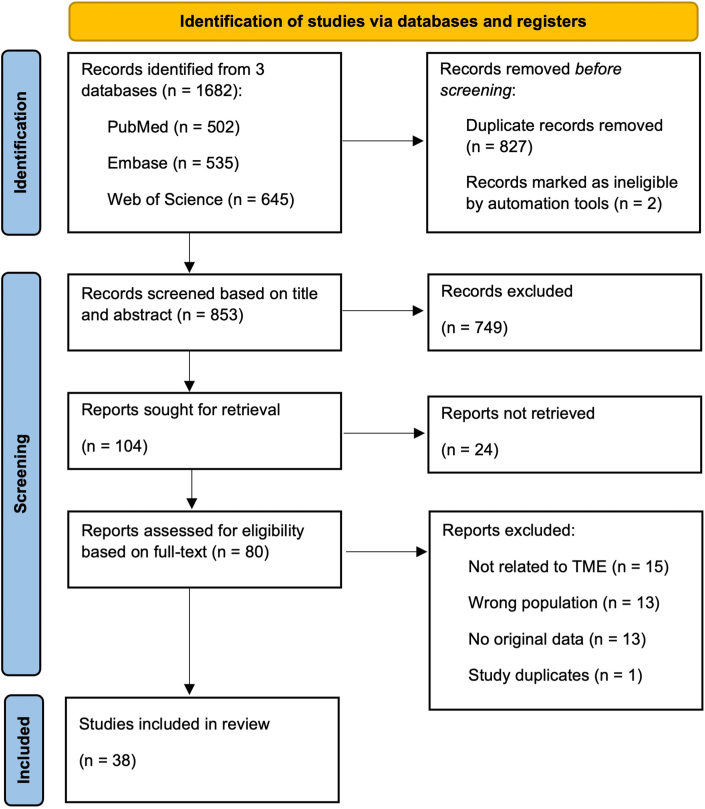
**PRISMA flow diagram.** PRISMA, Preferred Reporting Items for Systematic Reviews and Meta-Analysis.

A total of 1682 articles were retrieved through a literature search. After removing duplicates, 853 articles remained for titles and abstracts review, but 749 of these did not meet the inclusion criteria. Consequently, the remaining 104 articles were screened in full text, of which 38 were included in the final qualitative synthesis (Figure 2). To summarize our findings, we synthesized and analyzed the extracted data to provide an overview of the current state of knowledge on TME of OTRs-cSCC and RDEB-cSCC. We divided the TME into 2 major complementary components: the tumor immune microenvironment (defined specifically by immune cells and associated factors) and the stromal compartment (characterized, for example, by ECM remodeling). Together, these components shape tumor progression and response to therapy. Below, we have categorized the results into 4 main topics: (i) the tumor immune microenvironment in OTRs-cSCC (Table 1); (ii) the tumor immune microenvironment in RDEB-cSCC (Table 2); (iii) tissue damage, COL7 loss, and ECM remodeling in RDEB-cSCC (Table 3); and (iv) ECM remodeling, neoangiogenesis, and cancer cell migration in OTRs-cSCC (Table 4).

**Table 1:** The Tumor Immune Microenvironment in OTRs-cSCC

Author Name and Publication Year	Biomarkers	Study Population	Methods	Main Findings
Dany et al, 2023	LAG-3	Normal skin (n = 5) cSCC comp good (n = 4) cSCC comp bad (n = 5) cSCC transplant good (n = 10) cSCC transplant bad (n = 5)	NanoString technology	LAG-3 mRNA expression was approximately 5-fold higher in immunocompetent-associated cSCC than in transplant-associated cSCC (*P* < .05).
Frazzette et al, 2020	CD8+ T cells	cSCC (n = 5) OTRs-cSCC (n = 6)	FC Single-cell sequencing	A decrease in CD8+ cytotoxic and naïve TILs was observed in TSCC, with regulatory TILs present in similar amounts.
Strobel et al, 2018	CD4, CD8, CD20, CD68	cSCC (n = 18) OTRs-cSCC (n = 20)	IHC	The density of CD4+ T cells, CD8+ T cells, and CD20+ tumor-infiltrating B cells in OTRs-cSCC was significantly reduced compared with that in ICPs.
Crespo et al, 2017	CD8+ T, CD20+ B, CD56+ NK, FOXP3+ Tregs	cSCC (n = 25) OTRs-cSCC (n = 59)	IHC	Intratumor infiltrates and tumor-specific T-cell responses were both lower in OTRs than in ICPs.
Abikhair et al, 2016	IL-22RA1	cSCC (n = 9) OTRs-cSCC (n = 7)	RT-PCR	cSCC tissue from OTRs showed increased expression of IL-22RA1.
Feldmeyer et al, 2016	FOXO1, OX40, CD3, CD4, CD8, FOXP3	cSCC (n = 10) OTRs-cSCC (n = 9)	nCounter IHC	Increased FOXO1 and OX40 expression as well as upregulation of Treg gene expression were found in OTRs-cSCC compared with those in ICPs.
Sandvik et al, 2015	DC subsets, macrophages, FOXP3+ T cells	cSCC (n = 15) OTRs-cSCC (n = 15)	IHC	OTRs-cSCC showed a significant reduction in CD11c+ mDC and FOXP3+ T cells, whereas pDC, Langerhans cells, and macrophages were present at similar levels as in common cSCC.
Zhang et al, 2013	IL-22, IL-22R, IFN-γ, CD4, CD8, FOXP3, IL-4	cSCC (n = 10) OTRs-cSCC (n = 10)	IHC RT-PCR	TSCC was characterized by a higher proportion of IL-22–producing CD8+ T cells and a lower percentage of IFN-γ–producing CD4+ T cells compared with those in cSCC.
Krynitz et al, 2010	CD3, CD4, CD8, CD56, CD20, CD138, CD68,	cSCC (n = 14) OTRs-cSCC (n = 7)	IHC	The peritumoral immune infiltrate of OTRs-cSCC differed in cellular composition but not in cell density compared with those of ICPs.
Kosmidis et al, 2010	CD3, CD4, CD8, FOXP3, IFN-γ, IL-4, TGFβ	cSCC (n = 43) OTRs-cSCC (n = 42)	IHC RT-PCR	CD4 and IFN-γ mRNA expressions were markedly reduced in OTRs-cSCC. OTRs-cSCC exhibited a decline in Tregs, as indicated by lower FOXP3 and TGFβ mRNA levels.
Mühleisen et al, 2009	CD3, CD4, CD8, FOXP3, CD123	cSCC (n = 43) OTRs-cSCC (n = 42)	IHC	OTRs demonstrated markedly lower percentages of CD3+ and CD8+ cells than ICPs, with no notable difference in CD4+ cell proportions. A significant reduction in FOXP3+ Tregs was observed in OTRs-cSCC (*P* = .048).

Abbreviations: cSCC, cutaneous squamous cell carcinoma; DC, dendritic cell; FC, flow cytometry; ICP, immunocompetent patient; IHC, immunohistochemistry; mDC, myeloid dendritic cell; OTR, organ transplant recipient; OTRs-cSCC, cutaneous squamous cell carcinoma in organ transplant recipient; pDC, plasmacytoid dendritic cell; TIL, tumor-infiltrating lymphocyte; Treg, regulatory T cell; TSCC, transplant-associated squamous cell carcinoma.

cSCC comp good denotes cSCC tumors in ICPs with good outcomes, cSCC comp bad denotes cSCC tumors in ICPs with bad outcomes, cSCC transplant good denotes cSCC tumors in transplant patients with good outcome, and cSCC transplant bad denotes cSCC tumors in transplant patients with bad outcome.

**Table 2:** The Tumor Immune Microenvironment in RDEB-cSCC

Author Name and Publication Year	Biomarkers	Study Population	Methods	Main Findings
Ragot et al, 2024	Citrullinated histone H3	Low-risk primary RDEB-cSCC (n = 7) High-risk primary RDEB-cSCC (n = 5) Low-risk recurrent RDEB-cSCC (n = 16) High-risk recurrent RDEB-cSCC (n = 10)	IHC	In high-risk primary RDEB-cSCC, the TME displayed an increased neutrophil-to-lymphocyte ratio along with an increased proportion of NET marker, citrullinated histone H3.
Rafei-Shamsabadi et al, 2024	IDO, PD-1, PD-L1, TIM-3, LAG-3, CD4, CD8, CD68	cSCC (n = 100) RDEB-cSCC (n = 30) KEB-cSCC (n = 22)	IHC	IDO and PD-L1 levels in tumor cells as well as PD-1 expression in the TME were significantly higher in RDEB-cSCC than in cSCC from ICPs and ISPs.
Nezhad et al, 2022	CFD	Primary nonmetastatic cSCC (n = 140) Metastatic cSCC (n = 70) cSCC metastases (n = 9) RDEB-cSCC (n = 16)	IHC	CFD immunostaining in tumor cells was more intense at the tumor edge in primary and metastatic cSCC, including RDEB-associated cSCC, compared with that in cSCC in situ, AK, and normal skin.
Filoni et al, 2022	CD4+, CD8+, TIM-3, HO-1	cSCC (n = 12) RDEB-cSCC (n = 12)	IHC	RDEB-cSCC showed reduced levels of CD4+ T helper cells, CD8+ cytotoxic T cells, and HO-1 expression but elevated TIM-3 expression compared with primary cSCC.
Filoni et al, 2020	CD3+, CD4+, CD8+, CD20+, CD68+	cSCC (n = 10) RDEB-cSCC (n = 5) OTRs-cSCC (n = 5)	IHC	RDEB-cSCC showed significantly lower levels of CD3+, CD4+, CD8+, CD20+, and CD68+ cells than primary or secondary cSCC. In contrast, these markers (except CD4+) did not differ between RDEB-cSCC and cSCC in OTRs.
Riihilä et al, 2020	C1r, C1s	cSCC (n = 115) RDEB-cSCC (n = 16)	IHC	Tumor cells in invasive sporadic cSCC and RDEB-associated cSCC exhibited higher C1r and C1s expression than cSCC in situ, AK, and normal skin.
Riihilä et al, 2017	C3, CFB	cSCC (n = 71) RDEB-cSCC (n = 11)	IHC	C3 and CFB expressions were specifically upregulated in cSCC and RDEB-cSCC compared with those in cSCC in situ, AK, and normal skin.
Riihilä et al, 2015	CFI	cSCC (n = 83) RDEB-cSCC (n = 7)	IHC	Invasive sporadic cSCC and RDEB-associated cSCC showed stronger tumor cell–specific CFI staining intensity than cSCC in situ, AK, and normal skin.

Abbreviations: AK, actinic keratosis; C1, complement component 1; C1r, complement component 1r; C3, complement component 3; CFI, complement factor I; CFB, complement factor B; CFD, complement factor D; cSCC, cutaneous squamous cell carcinoma; HO-1, heme oxygenase-1; ICP, immunocompetent patient; IDO, indoleamine 2,3-dioxygenase; IHC, immunohistochemistry; ISP, immunosuppressed patient; KEB, Kindler epidermolysis bullosa; NET, neutrophil extracellular trap; OTR, organ transplant recipient; OTRs-cSCC, cutaneous squamous cell carcinoma in organ transplant recipient; RDEB, recessive dystrophic epidermolysis bullosa; RDEB-cSCC, recessive dystrophic epidermolysis bullosa–cutaneous squamous cell carcinoma; TME, tumor microenvironment.

**Table 3:** Tissue Damage, Collagen VII Loss, and ECM Remodeling in RDEB-cSCC

Author Name and Publication Year	Biomarkers	Study Population	Methods	Main Findings
Illmer et al, 2023	miR-200b, E-cadherin, ITGB3	RDEB-cSCC (n = 2) RDEB-cSCC cell lines	IF FC sqRT-PCR WB	miR-200b-3p downregulated in RDEB-cSCC.
Dayal et al, 2021	TGFβ1, TGFβ SMAD	RDEB-cSCC cell lines Subcutaneous xenografts mice model	WB IHC	Exogenous TGFβ triggered canonical SMAD signaling and induced proliferative arrest in all RDEB-cSCC cases.
Lincoln et al, 2021	Periostin, α-SMA	cSCCIS (n = 25) LR-cSCC (n = 26) HR-cSCC (n = 38) RDEB-cSCC (n = 6) cSCC cell lines (SCC12, SCC25) RDEB-cSCC cell lines (EB2K)	IHC IF WB	Periostin expression increased progressively and significantly (*P* < .001) from cSCCIS to LR-cSCC, HR-cSCC, and RDEB-cSCC.
Twaroski et al, 2021	TGFβ1, TGFBI, α-SMA, fibronectin, vimentin, periostin, MMP2, MMP9	RDEB-cSCC cell lines	WB ELISA IF	TGFβ1 released by RDEB dermal fibroblasts promoted migration, invasion, and upregulation of EMT markers in an RDEB-cSCC cell line.
Liao et al, 2018	MMP9, MMP13, IL-8, TGFβ1, TGFβ3, α-SMA, decorin	*Col7a1*^−/−^ (RDEB) mice model RDEB patient-derived cSCC cells	IHC qPCR IF	In *Col7a1*^−/−^ mice, TGFβ signaling was activated, along with enhanced collagen fibril deposition and increased dermal levels of MMP9 and MMP13.
Föll et al, 2018	APCS, vitronectin, collagen XIV,	Low-risk cSCC (n = 5) Metastasizing cSCC (n = 13) RDEB-cSCC (n = 6)	IF Bioinformatic analysis	Whereas sporadic cSCC exhibited features of UV-induced damage, RDEB-cSCC was characterized by a higher rate of sequence variants and tumor initiation driven by tissue injury, inflammation, and dermal ECM remodeling.
Mittapalli et al, 2016	Collagen VII, TGFβ, α-SMA, vimentin, pSMAD2/3, collagen I, E-cadherin, LOX	Carcinogen-treated RDEB mice model 3D organotypic RDEB skin cultures	qPCR IF WB	Repeated injury and the structurally unstable dermis in RDEB together enhanced TGFβ bioavailability.
Martins et al, 2015	TGFβ, VEGF, TGFβRI, twist, fibronectin, α2β1 integrin, collagen VII	cSCC (n = 44) RDEB-cSCC (n = 47) Human RDEB-cSCC cell lines (EB2K and EB3K) Mice xenograft model	IHC WB qPCR IF	RDEB-cSCC showed elevated TGFβ signaling, higher VEGF levels, and an increased number of cross-cut blood vessels compared with sporadic cSCC.
Ng et al, 2012	α-SMA, vimentin, type V collagen, thrombospondin-1, type XII collagen	cSCC (n = 6) RDEB-cSCC (n = 8)	WB IF IHC	Restoring wild-type *COL7A1* in RDEB fibroblasts led to reduced expression of type XII collagen, thrombospondin-1, and Wnt-5A; suppressed tumor cell invasion in organotypic models; and limited tumor growth in vivo.
Knaup et al, 2011	TGFβ	RDEB cell lines RDEB-cSCC cell lines (SCCRDEB4)	RT-PCR	Elevated levels of TGFβ1 and several genes under the control of TGFβ was found in RDEB and RDEB-cSCC cell lines.
Kivisaari et al, 2010	MMP7, CD44v3, HB-EGF	cSCC (n = 20) RDEB-cSCC (n = 60)	IHC WB	HB-EGF was not detected in tumor cells where MMP7 and CD44v3 were colocalized, with its absence being more prominent in RDEB-associated cSCC compared with that in non-EB cSCC.
Martins et al, 2009	MMP2, E-cadherin, N-cadherin, vimentin, αvβ3 integrin, collagen VII	RDEB-cSCC (n = 2) 3D organotypic skin model	IF	Collagen type VII loss enhanced migration and invasion in RDEB-cSCC, influenced cell differentiation, and facilitated EMT.
Kivisaari et al, 2008	MMP7, MMP13, MMP9, E-cadherin, syndecan-1	cSCC (n = 61) RDEB-cSCC (n = 25)	IHC	MMP7 was present in all RDEB-associated cSCCs, specifically in tumor cells at the invasive front, where E-cadherin and syndecan-1 were notably reduced or lost. Its staining intensity was significantly higher in RDEB-associated cSCC than in sporadic cSCC.

Abbreviations: 3D, 3-dimensional; α-SMA, α-smooth muscle actin; APCS, amyloid p component serum; CD44v3, CD44 variant 3; cSCC, cutaneous squamous cell carcinoma; cSCCIS, cutaneous squamous cell carcinoma in situ; ECM, extracellular matrix; EMT, epithelial–mesenchymal transition; FC, flow cytometry; HB-EGF, heparin-binding epidermal GF; HR-cSCC, high-risk cutaneous squamous cell carcinoma; IF, immunofluorescence; IHC, immunohistochemistry; LOX, lysyloxidase; LR-cSCC, low-risk cutaneous squamous cell carcinoma; MMP, matrix metalloproteinase; RDEB, recessive dystrophic epidermolysis bullosa; RDEB-cSCC, recessive dystrophic epidermolysis bullosa–cutaneous squamous cell carcinoma; sqRT-PCR, semiquantitative RT-PCR; WB, western blot.

**Table 4:** ECM Remodeling, Neoangiogenesis, and Cancer Cell Migration in OTRs-cSCC

Author Name and Publication Year	Biomarkers	Study Population	Methods	Main Findings
Verneuil et al, 2015	CD133, vimentin, snail, slug, E-cadherin	OTRs-cSCC (n = 3) OTRs-AK (n = 3)	IF ddPCR	In cSCC, donor-derived stem cells expressed EMT markers such as vimentin, snail, and slug, whereas these markers were absent in AK.
Gutiérrez-Dalmau et al, 2010	TGFβ1, p53, mTOR, phosphorylated mTOR, phosphorylated p70S6K	cSCC (n = 24) OTRs-cSCC (n = 27)	IHC	cSCC in OTRs exhibited higher p53 expression and stronger TGFβ signaling than in ICPs, whereas levels of phosphorylated mTOR and phosphorylated p70S6K (Thr421/Ser424) were elevated in cSCC from ICPs.
Rival-Tringali et al, 2009	VEGF, FOXP3+ Tregs	cSCC prior to SRL (n = 15) cSCC under SRL (n = 11)	IHC	cSCC developing under SRL showed lower peritumoral vascularization and thickness and higher growth fraction and density of peritumoral Tregs.
Harradine et al, 2009	TGFβ1, TGFβ2, TGFβ3, TGFβRII, P-Smad2/3, P-Smad1/5/8	cSCC (n = 87) OTRs-cSCC (n = 96)	IHC	Both lesional and nonlesional tissues from OTRs showed significantly elevated P-Smad2 levels than those from ICPs (*P* ≤ .001).
Kuivanen et al, 2009	MMP1, MMP7, MMP8, MMP9, MMP13, MMP26	cSCC (n = 20) OTRs-cSCC (n = 20)	IHC	Cancer cells in the post-transplant group showed significantly stronger MMP26 expression than those in ICPs (*P* = .01), whereas MMP9 was more prominently expressed by stromal macrophages surrounding cSCC in ICPs (*P* = .02).
Boyd et al, 2009	MMP10, MMP12, MMP21	cSCC (n = 25) OTRs-cSCC (n = 25)	IHC	Stromal MMP10 expression was higher (*P* = .009) in cSCC than in OTRs-cSCC. Stromal fibroblasts of cSCC tended to express MMP21 more abundantly.

Abbreviations: AK, actinic keratosis; cSCC, cutaneous squamous cell carcinoma; ddPCR, droplet digital PCR; ECM, extracellular matrix; EMT, epithelial–mesenchymal transition; ICP, immunocompetent patient; IF, immunofluorescence; IHC, immunohistochemistry; MMP, matrix metalloproteinase; OTR, organ transplant recipient; OTRs-cSCC, cutaneous squamous cell carcinoma in organ transplant recipient; P-Smad, phosphorylated Smad; SRL, sirolimus; Treg, regulatory T cell.

### The tumor immune microenvironment in OTRs-cSCC

Recent research on OTRs-cSCC has increasingly focused on the tumor immune microenvironment, particularly the role of tumor-infiltrating lymphocytes (TILs). These immune cells infiltrate the TME, playing a dual role in cancer immunosurveillance: exerting antitumor effects while also contributing to tumor immune evasion. TILs encompass various subsets, including T cells, B cells, and NK cells, each with distinct functions within the TME (Bottomley et al, 2019; de Visser and Joyce, 2023; Zilberg et al, 2023). To investigate the role of TILs in OTRs-cSCC, commonly used techniques in the included studies involved immunohistochemistry, RT-PCR, and single-cell sequencing, enabling the detection and quantification of immune cell subsets in tumor samples. On the basis of immunohistochemical analyses, most studies found that OTRs-cSCC exhibited a lower density of peritumoral inflammatory infiltrates than cSCC in immunocompetent individuals. Specifically, the proportions of CD3+ T lymphocytes, CD4+ helper T cells, and CD8+ cytotoxic T lymphocytes were reduced in OTRs-cSCC (Crespo et al, 2017; Kosmidis et al, 2010; Krynitz et al, 2010; Mühleisen et al, 2009; Strobel et al, 2018; Zhang et al, 2013). Notably, Mühleisen et al (2009) further demonstrated that in OTRs, immune attenuation was already evident in the premalignant stage because intraepithelial lesions (AK/Bowen’s disease) showed a lower density of perineoplastic infiltrates and fewer CD3+ and CD8+ T lymphocytes than in immunocompetent patients. In addition, significantly lower numbers of CD20+ B cells, CD56+ NK cells, CD11c+ myeloid dendritic cells, and CD123+ plasmacytoid dendritic cells were observed (Crespo et al, 2017; Mühleisen et al, 2009; Sandvik et al, 2015; Strobel et al, 2018). Conversely, in some studies, the amounts of CD4+ T-cells, Langerhans cells, and macrophages were not different between OTRs and immunocompetent patients (Krynitz et al, 2010; Mühleisen et al, 2009; Sandvik et al, 2015). Nevertheless, Viac et al, 1992 demonstrated that OTRs already showed reduced epidermal CD1+ Langerhans cells in AK, similar to findings in invasive cSCC, indicating an early local immune dysfunction in the premalignant setting. Studies using single-cell sequencing confirmed similarly reduced proportions of CD8+ cytotoxic and naïve TILs in OTRs-cSCC compared with those in cSCC in immunocompetent patients (Frazzette et al, 2020).

Notably, findings regarding regulatory T cells (Tregs) in OTRs-cSCC have been inconsistent across studies. Tregs, which can be identified by the marker FOXP3, are a subset of T cells that play a crucial role in suppressing antitumor immune responses and allowing tumor cells to evade immune surveillance (Jang, 2008). Some immunohistochemical studies have found significantly lower proportions of FOXP3+ Tregs in OTRs-cSCC (Crespo et al, 2017; Kosmidis et al, 2010; Mühleisen et al, 2009; Sandvik et al, 2015). Although it was postulated that immunosuppressive drugs reduce the proportion of Tregs, which may partially weaken their protumor effects, these drugs also diminish the function of other antitumor immune cells, making it difficult for the immune system of OTRs to effectively eliminate tumor cells (Mühleisen et al, 2009; Sandvik et al, 2015). In contrast, one study found that FOXP3+ mRNA expression was strongly upregulated in OTRs-cSCC (Feldmeyer et al, 2016), whereas in yet another study, the numbers of regulatory and exhausted T cells were similar in both patient groups as analyzed by single-cell RNA sequencing (Frazzette et al, 2020). Interestingly, in OTRs who developed cSCC before and after conversion from calcineurin inhibitors (CNIs) to the mTOR (mammalian target of rapamycin) inhibitor sirolimus, a higher peritumoral Treg density was detected in OTRs-cSCC grown under sirolimus (Rival-Tringali et al, 2009). CNIs such as cyclosporine A (CSA) suppress IFN-γ–driven T helper 1 responses and favor IL-22–mediated protumor signaling, whereas mTOR inhibitors can partially restore tumor-specific T-cell responses and reduce cSCC recurrence despite increased Tregs (Table 5) (Abikhair et al, 2016; Crespo et al, 2017; Rival-Tringali et al, 2009).

**Table 5:** Differential Effects of Immunosuppressive Agents on Immune and Stromal Components of the TME in OTRs-cSCC

Immunosuppressive Agent	Key Effect on Immune TME in OTRs-cSCC	Key Effect on Stromal TME in OTRs-cSCC
Calcineurin inhibitors (eg, cyclosporine A, tacrolimus)	↓IFN-γ/Th1; shift toward CD8^+^ Tc22/IL-22 axis; regimen-dependent ↓Treg generation or function; reduced CD4 mRNA and T-BET in peritumoral skin of OTRs; ↓IL-17A.	↑ TGFβ signaling, ↑ stromal activation, fibrosis
mTOR inhibitors (eg, sirolimus, everolimus)	After conversion from CNI: rebound of tumor-specific T-cell responses; higher intratumoral FOXP3^+^ Tregs yet overall functional improvement in effector responses.	↓ peritumoral vascularization and tumor thickness; antiangiogenic and antifibrotic effects

Abbreviations: CNI, calcineurin inhibitor; cSCC, cutaneous squamous cell carcinoma; OTR, organ transplant recipient; OTRs-cSCC, cutaneous squamous cell carcinoma in organ transplant recipient; Th1, T helper 1; TME, tumor microenvironment; Treg, regulatory T cell.

Although Treg/CD8 ratio was measured as increased in OTRs-cSCC, IL-22–producing CD8+ T cells were significantly more abundant in OTRs-cSCC than in cSCC from immunocompetent individuals, as shown by RT-PCR (Zhang et al, 2013). The mean mRNA expression of IL-22 receptor subunit *IL22RA1* increased approximately 3-fold in OTRs-cSCC by RT-PCR (Abikhair et al, 2016). After IL-22 binds to its receptor, it promotes tumor cell proliferation and antiapoptosis by activating the signal transducer and activator of transcription 3 signaling pathway (Abikhair et al, 2016; Zhang et al, 2013). Therefore, the increased mRNA expression of IL-22 and IL-22R may accelerate tumor growth in transplant recipients. In addition, increased mRNA expressions of FOXO1 (a transcription factor necessary for full Treg activity) and OX40 (which promotes the activation and antitumor activity of effector T cells) in the T-cell infiltrate surrounding OTRs-cSCC have also been demonstrated (Feldmeyer et al, 2016). These findings may explain the heightened aggressiveness of OTRs-cSCC compared with that of cSCC in immunocompetent patients.

In the view of recent development on ICIs, differences in the expression of immune checkpoint molecules have also been studied. LAG-3 (lymphocyte activation gene 3) is expressed on tumor-infiltrating T lymphocytes and binds to various tumor antigens. This interaction inhibits antigen-specific effector T-cell secretion of IFN-γ, thereby restricting antitumor response. LAG-3 mRNA expression was approximately 5-fold higher in immunocompetent patients than in OTRs (Dany et al, 2023). Table 1 has summarized the findings on the tumor immune microenvironment in OTRs-cSCC.

### The tumor immune microenvironment in RDEB-cSCC

Several studies have detected aberrant composition of immune cell infiltrate in RDEB-cSCC using immunohistochemistry over the past decade (Table 2). In RDEB-cSCC, the number of CD3+ T cells, CD4+ T lymphocytes, CD8+ T lymphocytes, CD20+ B cells, and CD68+ macrophages was lower than in sporadic cSCC (Filoni et al, 2022, 2020; Rafei-Shamsabadi et al, 2024). However, when comparing RDEB-cSCC with OTRs-cSCC, the immunohistochemical expressions of CD3+, CD8+, CD20+, and CD68+ cells were not significantly different (Filoni et al, 2020). Moreover, Nezhad et al (2022), Riihilä et al (2020, 2017), and Riihilä (2015) verified that several tumor cell–specific complement components (C1r, C1s, C3, CFB, CFD, CFI) were overexpressed in invasive sporadic cSCC and RDEB-cSCC compared with those in cSCC in situ, AK, and normal skin, suggesting that these components play an important role in the growth process of both invasive sporadic cSCC and RDEB-cSCC.

Indoleamine 2,3-dioxygenase (IDO) helps tumors to achieve immune escape by degrading L-tryptophan to kynurenine, which promotes Treg differentiation and inhibits effector T-cell responses (Rafei-Shamsabadi et al, 2024). IDO and PD-L1 were more highly expressed in cSCC from patients with dystrophic EB than in cSCC from immunocompetent and immunosuppressed individuals, and PD-1 was highly expressed in the TME (Rafei-Shamsabadi et al, 2024). The T-cell exhaustion marker TIM-3 was higher expressed in RDEB-cSCC than in sporadic cSCC, and immunohistochemical expression of heme oxygenase-1 (HO-1) in the TME of RDEB-cSCC was markedly reduced compared with that in sporadic cSCC (Filoni et al, 2022). HO-1 is an inducible microsomal enzyme that breaks down heme to produce the antioxidants biliverdin and carbon monoxide, thereby exerting antioxidant and anti-inflammatory effects. The significant reduction of HO-1 in RDEB-cSCC may disrupt normal antioxidant and anti-inflammatory regulation, making the TME more susceptible to oxidative stress, thus promoting tumor growth and spread. In addition, higher neutrophil-to-lymphocyte ratios and elevated levels of the neutrophil extracellular trap marker citrullinated histone H3 have been identified in the TME of high-risk primary RDEB-cSCC compared with those in low-risk primary RDEB-cSCC, peritumoral skin, and nonlesional skin (Ragot et al, 2024).

### Tissue damage, COL7 loss, and ECM remodeling in RDEB-cSCC

It is hypothesized that the repetitive wound and scar formation in RDEB results in extensive fibrosis leading to a “tumor-prone” stromal microenvironment (Martins et al, 2009; Ng et al, 2012). The exact molecular mechanisms underlying this process are not known, but the loss of COL7 seems to lead to changes in the ECM that promote epithelial–mesenchymal transition (EMT) and cSCC invasion (Martins et al, 2009; Ng et al, 2012; Sistigu et al, 2017). EMT is an important process in which epithelial cells lose intracellular adhesion properties and acquire cell motility, thereby facilitating tumor invasion and metastasis. EMT is characterized by the disappearance of epithelial markers (eg, E-cadherin) and concurrent acquisition of mesenchymal markers (eg, N-cadherin, vimentin) in tumor cells (Sistigu et al, 2017; Twaroski et al, 2021).

It is postulated that canonical TGFβ signaling, in response to the loss of COL7, plays an important role in the creation of the permissive TME and subsequently cancer initiation and progression of RDEB-cSCC (Dayal et al, 2021; Knaup et al, 2011). The inherent structural instability of the RDEB dermis, combined with repeated injury, increases the bioavailability of TGFβ, primarily sourced from fibroblasts and inflammatory cells, which in turn promotes ECM production, cross-linking, dermal fiber thickening, and tissue stiffening (Dayal et al, 2021; Mittapalli et al, 2016) (Table 3). Compared with those in sporadic cSCC, increased microvascularization, TGFβ signaling, and VEGF expression were observed in RDEB-cSCC (Martins et al, 2016). Importantly, COL7 inhibits TGFβ signaling and angiogenesis in cSCC by interacting with α2β1 integrin in keratinocytes.

Whereas sporadic cSCC shows signs of UV damage, RDEB-cSCC is characterized by a higher rate of sequence variants, with tissue damage, inflammation, and subsequent dermal ECM remodeling acting as tumor-initiating factors (Föll et al, 2018). Regarding ECM remodeling, miR-200b-3p, a specific microRNA that maintains epithelial characteristics by inhibiting specific transcription factors, was found to be downregulated in RDEB-cSCC. Reintroducing miR-200b-3p into RDEB-cSCC cell lines with different EMT states by microRNA mimic transfection significantly enhanced epithelial characteristics and reduced mesenchymal traits (Illmer et al, 2023). Matrix metalloproteinases (MMPs), members of the endopeptidase family, promote the growth, invasion, and metastasis of cSCC by degrading the ECM and facilitating the spread of cancer cells into surrounding tissues. The immunohistochemical staining of MMP7 was significantly stronger in RDEB-cSCC than in sporadic cSCC and is highest in poorly differentiated tumors (Kivisaari et al, 2010; Kivisaari et al, 2008). In *Col7a1*^−/−^ mice, activation of TGFβ signaling was accompanied by an increase in collagen fibril deposition and elevated immunohistochemical expression of MMP9 and MMP13 (Liao et al, 2018). Furthermore, periostin levels were significantly elevated in RDEB-cSCC compared with those in sporadic cSCC. Periostin is expressed by RDEB fibroblasts and CAFs in the TME and plays a critical role in promoting EMT, angiogenesis, invasion, and metastasis in tumors, making it an important biomarker for assessing the prognosis of cSCC (Lincoln et al, 2021).

### ECM remodeling, neoangiogenesis, and cancer cell migration in OTRs-cSCC

Biomarkers specifically expressed in the stromal TME of OTRs-cSCC are shown in Table 4. Different immunosuppressive agents appear to exert distinct effects on the stromal microenvironment. CNIs such as CSA or tacrolimus are associated with enhanced TGFβ signaling and Smad2 activation in both tumor epithelial and stromal compartments, indicating a profibrotic and tumor-promoting milieu (Gutiérrez-Dalmau et al, 2010; Harradine et al, 2009). Consistently, immunohistochemical analyses revealed higher expression of phosphorylated Smad2 and stronger TGFβ intensity in OTRs-cSCC than in cSCC of immunocompetent patients (Harradine et al, 2009). Of note, similar alterations in TGFβ/Smad signaling have already been detected in the premalignant setting: elevated nuclear phosphorylated Smad2 levels were observed not only in invasive OTRs-cSCC but also in cSCC in situ and AK as well as in adjacent nonlesional skin, compared with those in immunocompetent individuals (Harradine et al, 2009). These findings indicate that TGFβ pathway activation precedes malignant transformation and is already present in early, premalignant stages of keratinocytic neoplasia under immunosuppression. In contrast, mTOR inhibitors (eg, sirolimus) exhibit antiangiogenic and antiproliferative effects, reflected by decreased peritumoral vascularization and reduced tumor thickness after conversion from CNI therapy (Rival-Tringali et al, 2009). These findings indicate that distinct immunosuppressive regimens differentially influence stromal activation and angiogenic remodeling within OTRs-cSCC (Table 5).

In the context of OTRs-cSCC, differences in immunohistochemical expression of MMPs have been shown compared with those in immunocompetent individuals. Notably, more MMP26-positive cancer cells from the post-transplantation group were detected compared with those from the immunocompetent group (Kuivanen et al, 2009). Conversely, a higher expression of MMP9, 10, and 21 in the stromal TME of cSCC of immunocompetent patients were detected (Boyd et al, 2009; Kuivanen et al, 2009).

Interestingly, donor-derived epithelial stem cells were identified in the basal layers and invasive areas of cSCC and in the basal layers of AK in kidney transplant recipients but not in the normal surrounding skin tissue using XY-FISH technology. This technique enables the detection of Y-chromosome–bearing cells in female recipients who had received male kidney transplants. These donor-derived stem cells expressed mesenchymal markers (vimentin, Snail, and Slug) in the invasive region of cSCC but not in AK. Simultaneously, the immunohistochemical expression of claudin-1 and desmoglein-1 (epithelial markers) was reduced or absent (Verneuil et al, 2015). Previously unreported, to our knowledge, this study confirms that donor-derived stem cells may migrate, engraft, and exhibit potential invasive capabilities within OTRs-cSCC, offering insight into the complexity of transplant-associated tumors. However, owing to the limited number of donor-derived stem cells detected, it cannot be concluded that these cells alone drive tumor growth.

## Discussion

On the basis of the literature, RDEB-cSCC is more driven by increased TGFβ signaling with associated ECM remodeling of the peritumoral stroma (Dayal et al, 2021; Mittapalli et al, 2016), whereas the TME of OTRs-cSCC is characterized by immune exhaustion and dysregulated Treg activity (Crespo et al, 2017; Kosmidis et al, 2010; Krynitz et al, 2010; Mühleisen et al, 2009; Sandvik et al, 2015; Strobel et al, 2018; Zhang et al, 2013).

However, there are also shared characteristics in both types of cSCC. In recent studies, it was shown that also an immunosuppressive TME, characterized by reduced immune cell infiltration and enhanced expression of inhibitory immunoregulatory proteins, favors tumor progression and metastasis in RDEB-cSCC (Filoni et al, 2022, 2020; Rafei-Shamsabadi et al, 2024). Interestingly, it is hypothesized that the aggressive behavior of OTRs-cSCC is not only facilitated by direct immunosuppression but also by the activation of the TGFβ signaling pathway in the TME (Gutiérrez-Dalmau et al, 2010; Harradine et al, 2009). Therefore, it seems that in both OTRs and patients with RDEB, a “tumor-prone” microenvironment is created by immune dysfunction and increased TGFβ signaling.

Whereas canonical TGFβ signaling in RDEB skin is associated with loss of COL7 and concomitant chronic wounding (Dayal et al, 2021; Knaup et al, 2011; Martins et al, 2016), immunosuppressive drugs may activate the TGFβ signaling axis in OTRs through a nonimmune-mediated pathway (Harradine et al, 2009; Hojo et al, 1999; Maluccio et al, 2003). More specifically, we hypothesize that immunosuppressive drugs induce the conversion of dermal fibroblasts to CAFs through a TGFβ-dependent mechanism, leading to oncogenic changes in the TME of OTRs-cSCC. Importantly, this TGFβ-mediated phenotypic switch to CAFs is a crucial mechanism in the modulation of the ECM and, eventually, creation of a cancer-supporting TME in RDEB-cSCC (Martins et al, 2009; Ng et al, 2012). Our hypothesis is supported by findings that the CNI tacrolimus induces phenotypic fibroblast transition through TGFβ signaling in a renal fibrosis model and enhances cell invasion in a fibroblast–cSCC spheroid coculture in vitro, suggesting that tacrolimus-activated fibroblasts exert tumor-promoting effects on cSCC cells (Kumah et al, 2024; Ume et al, 2023). CAFs are important stromal modulators of the TME, which exert immunosuppressive and cancer-promoting effects by inducing antigen-specific deletion of cytotoxic T cells and accumulation of protumoral macrophages (Lakins et al, 2018; Takahashi et al, 2017). That could mean that immunosuppressive drugs might enforce tumor progression, not only by directly inhibiting immune cell function in OTRs-cSCC but also indirectly through TGFβ-mediated activation of CAFs in the stromal TME. However, the impact of immunosuppressive drugs on ECM remodeling as well as the role of TGFβ warrant further investigation—particularly the molecular mechanisms through which CNI may induce TGFβ activation and promote a phenotypic switch in dermal fibroblasts.

Moreover, understanding how EMT contributes to the aggressiveness of both OTRs-cSCC and RDEB-cSCC is crucial to elucidate in future research. TGFβ seems to be an important regulator and driver in the induction of EMT in RDEB-cSCC (Martins et al, 2009; Santibanez et al, 2018). In addition, MMPs, regulated by TGFβ, may play a significant role in EMT in both groups because increased immunohistochemical expression of different types of MMPs was seen in RDEB-cSCC and OTRS-cSCC (Boyd et al, 2009; Kivisaari et al, 2010, 2008; Kuivanen et al, 2009; Liao et al, 2018). However, more evidence about the exact interplay between TGFβ, MMPs, and other ECM components is needed for defining specific stromal biomarkers for the assessment of aggressive behavior and, potentially, prognosis in both OTRS-cSCC and RDEB-cSCC.

As previously noted, immunosuppressive therapy directly inhibits immune cell function in OTRs-cSCC; however, the composition of the tumor immune microenvironment appears to be strongly influenced by the specific type of therapy, which might explain the controversial results of previous studies on this subject. In particular, the regulation of FOXP3+ Tregs is differentially affected by CNIs and mTOR inhibitors. Multiple clinical and pathological studies have demonstrated that CNIs, such as CSA, significantly increase the risk of developing and recurring cSCC in OTRs (Crespo et al, 2017; Rival-Tringali et al, 2009). In contrast, switching to mTOR inhibitors, such as sirolimus, has been associated with a reduced incidence and invasiveness of cSCC, along with favorable immunological remodeling—characterized by the restoration of tumor-specific T-cell responses and increased Treg infiltration (Crespo et al, 2017; Rival-Tringali et al, 2009). These findings suggest that sirolimus may possess both immunomodulatory and antitumor properties.

Beyond its suppressive effects on Tregs, CSA appears to promote tumor progression through additional cytokine-mediated mechanisms. A pivotal study by Abikhair et al (2016) established a compelling link between CSA administration and activation of the IL-22 axis in cSCC. In vitro, CSA enhanced IL-22–mediated proliferation, migration, and invasion of cSCC cells. Moreover, in a UVB-induced murine model of cSCC, IL-22 blockade significantly reduced tumor number and burden (Abikhair et al, 2016). These results suggest that CSA not only compromises tumor surveillance by inhibiting calcineurin and suppressing Treg but also fosters a tumor-promoting microenvironment by amplifying IL-22 signaling pathways. Although increased Treg levels induced by mTOR inhibitors may reflect improved immune regulation, the precise functional phenotype and impact of these Tregs on tumor progression remain uncertain. Whether Treg enrichment promotes immune tolerance and tumor evasion or instead reflects immune normalization within the TME remains controversial. Future studies should focus on dissecting the suppressive function, antigen specificity, and broader immunoregulatory roles of Tregs under different immunosuppressive regimens in OTRs-cSCC, ideally in conjunction with analyses of effector T-cell function and antigen-presenting cell activity.

On the basis of the aforementioned findings and hypotheses, targeting the TGFβ signaling pathway may represent a promising therapeutic strategy to mitigate cSCC progression in both high-risk patient groups. This theory is supported by the finding that the angiotensin receptor blocker losartan was found to ameliorate disease burden in children with RDEB by inhibiting TGFβ activity (Kiritsi et al, 2024; Nyström et al, 2015). However, the paradoxical role of TGFβ signaling in oncogenesis, exhibiting both pro and antitumor proliferative functions (Colak and ten Dijke, 2017), poses a challenge in the implementation of TGFβ inhibitors as a cancer therapy owing to a potential double-edged sword effect (Dayal et al, 2021; Guttmann-Gruber and Piñón Hofbauer, 2021). A better understanding of the molecular mechanisms underlying the heterogenous response to TGFβ signaling inhibition in the stromal TME is essential for the optimization of TGFβ inhibitors in the treatment of OTRs-cSCC and RDEB-cSCC.

Several studies have shown that ICIs are promising for the treatment of advanced cSCC (Koch Hein et al, 2023; Stevenson et al, 2017). However, significant challenges remain in terms of efficacy and safety in OTRs-cSCC and RDEB-cSCC. Specifically, for OTRs-cSCC, only the expression of the immune checkpoint molecule LAG-3 has been studied (Dany et al, 2023), although OTRs were included in studies where the expression of other key immune checkpoint molecules has been examined in cSCC from immunocompetent and immunosuppressed patients. It was shown that PD-L1 expression was not significantly different between both groups, suggesting that PD-1 inhibitors could be an efficient therapeutic intervention in OTRs-cSCC (Varki et al, 2018). Interestingly, retrospective studies demonstrated that PD-1 inhibitors were associated with improved cancer outcomes in OTRs-cSCC (Murakami et al, 2021; Tsung et al, 2021). Nevertheless, one serious concern with the use of ICIs is the risk of organ rejection due to disrupting immune tolerance. The type and number of immunosuppressants may influence the probability of organ rejection, but additional studies are needed to determine efficient and safe immunotherapeutic strategies in OTRs-cSCC (Kawashima et al, 2022).

It is surmised that the high expression of the immune checkpoint molecules PD-1, PD-L1, and TIM-3 is an important mechanism in the development of an immunosuppressive TME in RDEB-cSCC. Moreover, these markers could be promising candidate targets for immunotherapy in this patient group (Rafei-Shamsabadi et al, 2024). Recent reports stated that especially cemiplimab induced a durable clinical response in patients with severe RDEB with metastasised cSCC (Duong et al, 2021; Khaddour et al, 2020; Robertson et al, 2021; Trefzer et al, 2023). However, PD-1 inhibition in combination with other therapeutic targets, such as IDO inhibitors, could improve treatment efficacy in RDEB-cSCC (Trefzer et al, 2023). Further characterization of the tumor immune microenvironment of RDEB-cSCC could not only shed light on the role of immunological mechanisms in the tumorigenesis but could also help to guide therapeutic decisions.

### Limitations

One limitation of this scoping review is that we only focused on the TME of OTRs-cSCC and RDEB-cSCC and did not describe other (high-risk) cSCC groups, even excluding studies on patient groups with different causes of immunosuppression. However, we deliberately limited our literature search to articles that strictly met the inclusion criteria to specifically investigate similarities and differences in the TME between OTRs-cSCC and RDEB-cSCC. Moreover, we compared the TME of both groups with findings in sporadic cSCC. Although immune evasion and ECM remodeling are also crucial for cancer progression in sporadic cSCC, we detected that in OTRs-cSCC and RDEB-cSCC, several complex tumor-promoting effects in the TME may be enhanced, favoring the development of aggressive cSCC in both high-risk groups.

### Concluding remarks and future perspectives

This scoping review highlights the unique features of the TME in RDEB-cSCC and OTRs-cSCC. We conclude that the TME in both high-risk groups is characterized by immune dysregulation and TGFβ signaling, suggesting overlapping tumorigenic mechanisms and potential shared therapeutic targets.

Several knowledge gaps persist in our understanding of the TME in OTRs-cSCC and RDEB-cSCC. Emerging techniques such as single-cell analyses and spatial transcriptomics offer promising avenues for profiling specific cell populations within the TME, providing locational context. Specifically, spatial transcriptomics could be performed to analyze immune cell populations in the TME after ICI treatment, aiding in the identification of potential immune checkpoints and the prediction of therapeutic efficacy in both high-risk groups. Therefore, these advanced methods could shed more light on the various intrinsic and extrinsic processes of the TME, which could eventually lead to a better understanding of the aggressive nature of RDEB-cSCC and OTRs-cSCC and the optimization of therapeutic interventions.

## Materials and Methods

This scoping review was performed according to the PRISMA-ScR (Preferred Reporting Items for Systematic Reviews and Meta-Analysis Extension for Scoping Reviews) guidelines (Page et al, 2021; Tricco et al, 2018). A systematic literature search was conducted in PubMed, Embase, and Web of Science on June 28, 2024, using a search strategy (Table 6) with no date restrictions. Duplicate literature was deleted using the automatic and manual duplicate removal function of Endnote 21 (Clarivate Analytics, London, United Kingdom).

**Table 6:** Search Strategy Used per Database

Database	Search Strategy Search Date (June 28, 2024)	Results
PubMed	("Carcinoma, Squamous Cell"[Mesh] OR "Cutaneous Squamous Cell Carcinoma∗" [tiab] OR "cSCC"[tiab] OR "Squamous Cell Carcinoma∗"[tiab] OR "SCC"[tiab]) AND ("Epidermolysis Bullosa"[Mesh] OR "EB"[tiab] OR "Recessive Dystrophic Epidermolysis Bullosa"[tiab] OR "RDEB"[tiab] OR "Organ Transplantation"[Mesh] OR "Organ Transplant Recipient∗"[tiab] OR "OTR"[tiab] OR "Immunosuppressed"[tiab] OR "Immunocompetent"[tiab]) AND ("Tumor Microenvironment"[Mesh] OR "Tumor Microenvironment"[tiab] OR "TME"[tiab] OR "Immune Microenvironment"[tiab] OR "Stromal Microenvironment"[tiab] OR "Extracellular Matrix"[tiab] OR "Epithelial-Mesenchymal Transition"[tiab] OR "EMT"[tiab] OR "Type VII Collagen"[tiab] OR "Transforming Growth Factor∗"[tiab] OR "TGFβ"[tiab] OR "Cancer-Associated Fibroblast∗"[tiab] OR "Immune Cell∗"[tiab] OR "T Cell∗"[tiab] OR "Matrix Metalloproteinase∗"[tiab] OR "MMP"[tiab] OR "Complement"[tiab] OR "Biomarker∗"[tiab] OR "Gene Expression"[tiab] OR "Mutation∗"[tiab] OR "Pathogenesis"[tiab])	502
Embase	('squamous cell skin carcinoma'/exp OR ('cutaneous squamous cell carcinoma∗' OR 'cscc' OR 'squamous cell carcinoma∗' OR 'scc'):ab,ti,kw) AND ('epidermolysis bullosa'/exp OR 'organ transplantation'/exp OR ('eb' OR 'recessive dystrophic epidermolysis bullosa' OR 'rdeb' OR 'organ transplant recipient∗' OR 'otr' OR 'immunosuppressed' OR 'immunocompetent'):ab,ti,kw) AND ('tumor microenvironment'/exp OR ('tumor microenvironment' OR 'tme' OR 'immune microenvironment' OR 'stromal microenvironment' OR 'extracellular matrix' OR 'epithelial-mesenchymal transition' OR 'emt' OR 'type vii collagen' OR 'transforming growth factor∗' OR 'tgfβ' OR 'cancer-associated fibroblast∗' OR 'immune cell∗' OR 't cell∗' OR 'matrix metalloproteinase∗' OR 'mmp' OR 'complement' OR 'biomarker∗' OR 'gene expression' OR 'mutation∗' OR 'pathogenesis'):ab,ti,kw) NOT 'conference abstract'/it	535
Web of Science	TS=("Cutaneous Squamous Cell Carcinoma∗" OR "cSCC" OR "Squamous Cell Carcinoma∗" OR "SCC") AND TS=("Epidermolysis Bullosa" OR "EB" OR "Recessive Dystrophic Epidermolysis Bullosa" OR "RDEB" OR "Organ Transplant Recipient∗" OR "OTR" OR "Immunosuppressed" OR "Immunocompetent") AND TS=("Tumor Microenvironment" OR "Tumor Microenvironment" OR "TME" OR "Immune Microenvironment" OR "Stromal Microenvironment" OR "Extracellular Matrix" OR "Epithelial-Mesenchymal Transition" OR "EMT" OR "Type VII Collagen" OR "Transforming Growth Factor∗" OR "TGFβ" OR "Cancer-Associated Fibroblast∗" OR "Immune Cell∗" OR "T Cell∗" OR "Matrix Metalloproteinase∗" OR "MMP" OR "Complement" OR "Biomarker∗" OR "Gene Expression" OR "Mutation∗" OR "Pathogenesis")	645

The remaining studies were screened for relevance by 2 independent authors (WW and CH) using titles and abstracts in the web-based tool Rayyan (Rayyan Systems, https://rayyan.ai/). In cases of uncertainty about the appropriateness of an article, the full-text review was evaluated, and consensus was reached by discussion. Articles were screened on the basis of the following inclusion criteria: (i) published in English, (ii) available in full text, and (iii) focused on the TME of cSCC in OTRs and/or EB-associated cSCC. Articles were excluded if they (i) had no original data (conference abstracts, reviews, commentaries, editorials, brief reports, and meta-analyses), (ii) had incorrect study populations, or (iii) had no data related to TME.

## Ethics Statement

No human or animal studies were conducted for this manuscript.

## Data Availability Statement

No datasets were generated or analyzed; data sharing is not applicable.

## ORCIDs

Wandong Wang: http://orcid.org/0009-0006-6578-7192

Clara Harrs: http://orcid.org/0000-0002-0034-911X

Maria C. Bolling: http://orcid.org/0000-0003-2086-9363

Barbara Horváth: http://orcid.org/0000-0001-8559-3674

Gilles F. H. Diercks: http://orcid.org/0000-0001-8053-216X

Emőke Rácz: http://orcid.org/0000-0001-5119-6451

## Conflict of Interest

The authors state no conflict of interest.
